# The [Mo_6_Cl_14_]^2−^ Cluster is Biologically Secure and Has Anti-Rotavirus Activity In Vitro

**DOI:** 10.3390/molecules22071108

**Published:** 2017-07-05

**Authors:** Edgardo Rojas-Mancilla, Alexis Oyarce, Viviana Verdugo, Cesar Morales-Verdejo, Cesar Echeverria, Felipe Velásquez, Jonas Chnaiderman, Fernando Valiente-Echeverría, Rodrigo Ramirez-Tagle

**Affiliations:** 1 Departamento de Ciencias Químicas y Biológicas, Universidad Bernardo O Higgins, General Gana 1702, Santiago 8370854, Chile; edgardo.rojas@ubo.cl; 2 Escuela de Tecnología Médica, Universidad Bernardo O Higgins, General Gana 1702, Santiago 8370854, Chile; alexis.oyarce@hotmail.com (A.O.); viviana.verdugo@live.com (V.V.); 3 Centro Integrativo de Biología y Química Aplicada, Universidad Bernardo O Higgins, General Gana 1702, Santiago 8370854, Chile; cesar.echeverria@ubo.cl (C.M.-V.); cesar.morales@ubo.cl (C.E.); 4 Instituto de Ciencias Biomédicas, Programa de Virología, Universidad de Chile, Avda, Independencia 1027, Independencia 8380453, Chile; fe.velasquezs@gmail.com (F.V.); jchnaiderman@med.uchile.cl (J.C.); fvaliente@uchile.cl (F.-V.E.); 5 Facultad de Ingeniería, Ciencia y Tecnología, Universidad Bernardo O Higgins, Avenida Viel 1497, Santiago 8370993, Chile

**Keywords:** cluster, cell viability, hemolysis, red blood cells, albumin, rotavirus

## Abstract

The molybdenum cluster [Mo_6_Cl_14_]^2−^ is a fluorescent component with potential for use in cell labelling and pharmacology. Biological safety and antiviral properties of the cluster are as yet unknown. Here, we show the effect of acute exposition of human cells and red blood cells to the molybdenum cluster and its interaction with proteins and antiviral activity in vitro. We measured cell viability of HepG2 and EA.hy926 cell lines exposed to increasing concentrations of the cluster (0.1 to 250 µM), by 3-(4,5-dimethylthiazol-2-yl)-2,5-diphenyltetrazolium bromide (MTT) colorimetric assay. Hemolysis and morphological alterations of red blood cells, obtained from healthy donors, exposed to the cluster (10 to 200 µM) at 37 °C were analyzed. Furthermore, quenching of tryptophan residues of albumin was performed. Finally, plaque formation by rotavirus SA11 in MA104 cells treated with the cluster (100 to 300 µM) were analyzed. We found that all doses of the cluster showed similar cell viability, hemolysis, and morphology values, compared to control. Quenching of tryptophan residues of albumin suggests a protein-cluster complex formation. Finally, the cluster showed antiviral activity at 300 µM. These results indicate that the cluster [Mo_6_Cl_14_]^2−^ could be intravenously administered in animals at therapeutic doses for further in vivo studies and might be studied as an antiviral agent.

## 1. Introduction

The hexamolybdenum halide clusters are characterized by showing long emission lifetimes and electronic absorption spectra, mostly composed of intense ligand to metal charge transfer type transitions [1,2,3,4,5]. Thus, the chemistry of the hexamolybdenum halide clusters is particularly attractive due to their synthetic versatility, thermal stability, luminescence, and redox properties [6,7,8,9,10]. The molybdenum hexanuclear type [M_6_X_8_Y_6_] cluster and their derivatives have attracted increasing attention due to recent potential pharmacological applications [11,12]. Changes in their structure have offered a high degree of diversity that has proven useful for the development of new medicinal agents their improved potency and lesser toxicity [6,11], effects on in a wide range of cell types, in vitro, [13] and varying ligands, resulting in multiple components with diverse photophysical and redox properties [7]. Up to now, luminescent properties of cluster were known [2,4,5,7,14].

The potential use of luminescent clusters in the biomedical field depends on its biological safety evaluation, including studying cellular uptake and subcellular localization, cytotoxicity, and hemolytic properties [15,16,17]. Cell uptake depends on the interaction of the cluster with the cell membranes. Although the clusters may show cytotoxic effects, it is necessary to test if they produce possible cellular damage before applying these components to animal models [17]. In addition, red blood cells could be affected, their number reduced by hemolysis, causing a condition named anemia, which leads to a lower oxygen transport capacity [18,19].

Almost all drugs form complexes with proteins, mainly albumin, in blood circulation [20]. Quenching of fluorescence have been applied to evaluate the interaction of clusters with proteins like albumin [21]. Quenching of fluorescence of albumin by complexes is either by a dynamic or static mechanism. Dynamic quenching refers to a process in which fluorophore and the quencher contact during the transient existence of the excited state, while static quenching refers to fluorophore-quencher complex formation. Dynamic quenching can be differentiated from static quenching by their temperature dependence. Dynamic quenching depends on the diffusion coefficient. Hence, an increase in temperature results in a higher diffusion coefficient and the bimolecular quenching constant is expected to increase with increased temperature. In contrast, in static quenching, increased temperature is likely to decrease the fluorophore-quencher stability constant resulting in lower values of quenching constants [21,22].

In the past, metal based components have received higher attention due to their potential to offer new therapies for health problems, like viral infections [23,24]. In this issue, polyoxometalate clusters, which are relatively similar to hexamolybdenum halide clusters, have been widely tested for antiviral activity in vitro, mainly against human immunodeficiency virus (HIV) and respiratory syncytial virus (RSV). Some clusters showed antiviral activity and low cell toxicity, opening a new avenue in virus research [24]. The mechanisms involved seems to be multiple, including inhibition of reverse transcriptase enzymes and blocking surface proteins of the virus [25]. Rotaviruses are the main cause of dehydrating diarrhea in children under five years old, requiring an effective therapy [26,27]. Considering that the molybdenum halide cluster presents a relatively close structure with other molybdenum clusters with anti-rotavirus activity, we hypothesize that the cluster [Mo_6_Cl_14_]^2−^ could show anti-rotavirus activity in vitro.

To establish whether cluster administration can induce toxic effects and/or hemolysis, HepG2, EA.hy926, and human red blood cells were exposed to different doses of the cluster in vitro and evaluated for possible effects on cell viability, hemolysis, and morphological alterations [16]. Furthermore, to evaluate a potential biomedical application of the cluster, cell labelling and antiviral activity were assessed. These biological in vitro studies, therefore, allow for the assessment of potential cell damage and applications in vivo, reducing the risk of systemic damage.

## 2. Results and Discussion

Acute exposition to the cluster induces low effects in both cell viability and hemolysis, and the cluster apparently forms a complex with albumin in solution. The anticancer activity of the cluster were tested in HepG2 and EA.hy926 cells. The targeting efficacy of the cluster showed higher toxicity and accumulation in both lines cells. The HepG2 was employed in the assays because it retains better hepatic characteristics, has been widely used for cytotoxicity studies [28,29], and, moreover, EA.hy926 is one of the most frequently used as a model of potential antitumor and antivascular effects [30]. Furthermore, the cluster showed cell labelling and antiviral properties.

### 2.1. Cell Uptake and Viability

**Figure 1:**
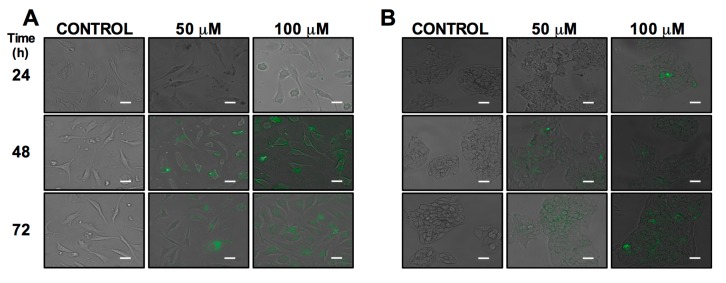
Cell uptake of the molybdenum cluster, and morphological changes in HepG2 and EA.hy926 cells. HepG2 (**A**) and EA.hy926 (**B**) cells were treated with the cluster for 24, 48 and 72 h. Pictures were taken under phase-contrast and epifluorescent microscope, showing that the cluster is incorporated in the cells and accumulated in the cytoplasm. Pictures show representative phase-contrast images from at least three separate experiments exposed to vehicle (control), or the cluster (50 and 100 μM). Bar scale represents 30 μm.

Cell uptake and morphological changes were observed in HepG2 and EA.hy926 human cells after 24, 48 and 72 h of cluster exposition (50 and 100 μM). HepG2 and EA.hy926 cells treated for 24 h did not show accumulation of the cluster at 50 and 100 μM (Figure 1A,B), while that cells treated for 48 and 72 h showed cluster uptake without morphological changes in both doses, and a uniform distribution of the cluster was observed in the cytoplasm and was not observed at the nuclear level in both cells (Figure 1A,B). The cluster is an anionic nanoparticle that could interact with the positive charges of the membrane proteins and affect the uptake pathway for endocytosis to the cell [31,32]. The anionic clusters are more likely to use caveolae-dependent endocytosis [33].

**Figure 2:**
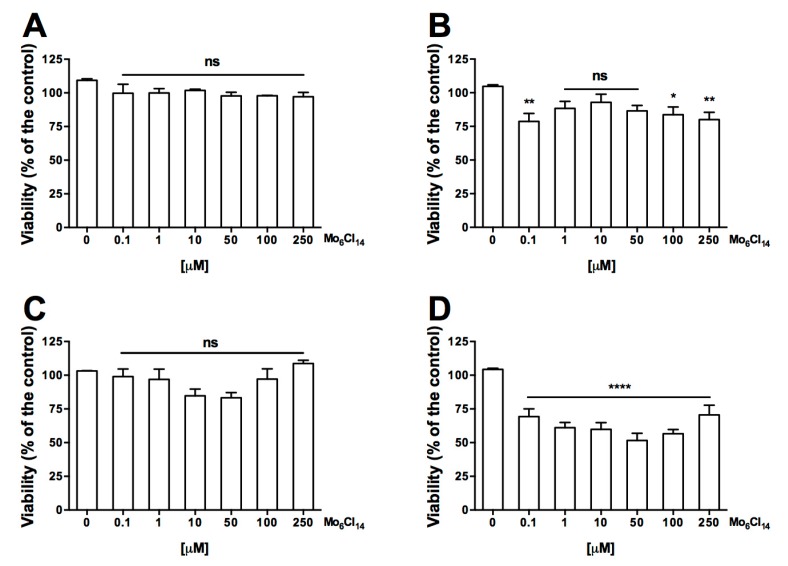
Effect of the cluster on the growth of HepG2 and EA.hy926 cells. The HepG2 (**A**,**B**) and EA.hy926 (**C**,**D**) were treated with increasing concentrations (0–250 μM) for 48 h (**A**,**C**) and 72 h (**B**,**D**). Cell viability was measured using the 3-(4,5-dimethylthiazol-2-yl)-2,5-diphenyltetrazolium bromide (MTT) colorimetric assay. Data is expressed as the mean ± SEM from three independent experiments, each performed in triplicate. Statistical differences were assessed by a one-way ANOVA (Kruskal-Wallis) followed by Dunn’s post hoc test. * *p* < 0.05, ** *p* < 0.01, **** *p* < 0.0001 compared with the condition 0 µM.

The cytotoxicity of molybdenum cluster was evaluated on HepG2 and EA.hy926 cell lines using the 3-(4,5-dimethylthiazol-2-yl)-2,5-diphenyltetrazolium bromide (MTT) colorimetric assay. All doses tested (0.1 to 250 µM) showed no toxicity on both HepG2 and EA.hy926 cells following 48 h of incubation (Figure 2). However, at 72 h of incubation, a cytotoxic effect was observed. Cytotoxicity in HepG2 cells was observed at a low dose (0.1 µM) and higher doses (100 and 250 µM), whereas in EA.hy926 cells, cytotoxicity was observed in all treatments. There is evidence that nanoparticles exert cytotoxicity through apoptosis; HepG2 [34] and endothelium [35] cells exposed to different nanoparticles have shown changes associated with apoptotic events. Future experiments are needed to elucidate whether cytotoxicity is related to apoptosis.

Several reports have described the antitumor effect of compounds synthesized from molybdenum. These clusters can be stimulated to generate the death of tumor cells [36] by inducing apoptosis through caspase activation [37], its antioxidant properties [38], or as a substitute for chemotherapy [39]. Molybdenum clusters have also shown photosensitizer properties, becoming promising components for photodynamic therapy, which requires agents that exert cytotoxic effects only under photoirradiation [40]. Future studies could be conducted to assess the cytotoxic effect of the cluster in tumor cells in vivo.

### 2.2. Hemolysis and Morphological Changes

**Figure 3:**
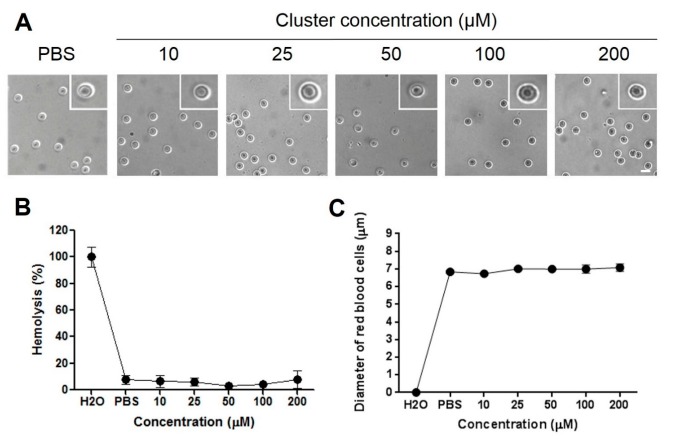
Hemolysis and morphology of human red blood cells after acute exposition (0 h) to increasing concentrations of the cluster. (**A**) Representative pictures of red blood cells at different cluster concentrations are shown. Exposition to 200 µM of the cluster induces slight crenation of red blood cells, compared to controls; (**B**) Hemolysis was similar to control (phosphate buffered saline (PBS)) through all concentrations; (**C**) The average size of erythrocytes is similar in all concentrations. Scale bar: 10 µm.

**Figure 4:**
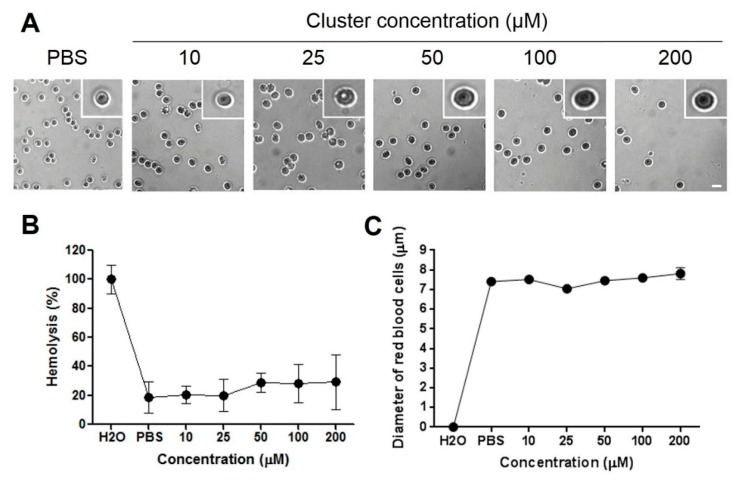
Hemolysis and morphology of human red blood cells after delayed exposition (24 h) to increasing concentrations of the cluster. (**A**) Representative pictures of red blood cells at different cluster concentrations are shown. Exposition to 200 µM of the cluster induces slight crenation of red blood cells, compared to controls; (**B**) Hemolysis was similar to control (PBS) through all concentrations; (**C**) The average size of erythrocytes is similar in all concentrations. Scale bar: 10 µm.

Acute exposition of human red blood cells to therapeutic doses of cluster did not induce acute (Figure 3B) or delayed (Figure 4B) hemolysis in vitro. Additionally, acute exposition to high doses of the cluster induced alterations in the morphology of red blood cells (Figure 3A and Figure 4A). Normal size of red blood cells ranged between 7 and 8 µM. Exposition to 10 to 200 µM of the cluster did not induce changes in average size, compared to controls, at 0 and 24 h (Figure 3C and Figure 4C). Red blood cells exposed to the cluster at 10 to 200 µM maintained the morphology at 0 and 24 h, showing only a slight crenation at the highest concentration (Figure 3A and Figure 4A).

We examined the hemolytic properties of increasing doses of the cluster in human red blood cells in vitro, finding a minimal hemolysis level at therapeutic and higher concentrations. This in vitro study needs to be corroborated by in vivo analysis of hemolytic properties, thus ensuring that administration in animals does not induce hemolytic anemia [41].

### 2.3. Protein Binding

**Figure 5:**
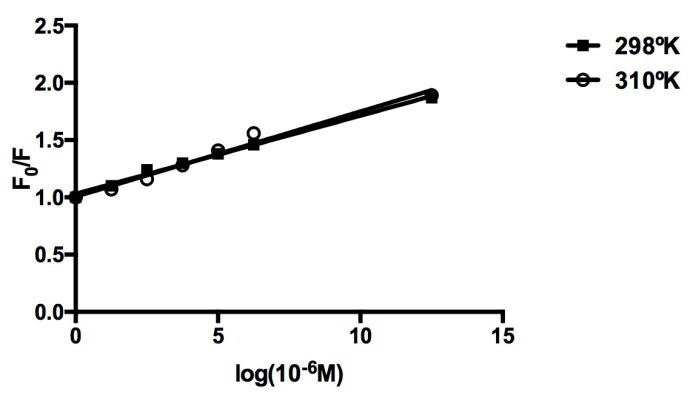
Plot of log(F_0_ − F)/F versus log[Q] for the Molybdenum [Mo_6_Cl_14_]^2−^ clusters-albumin system.

The tryptophan emission quenching experiments were carried out using human serum albumin in the presence of the cluster in order to investigate its interaction with proteins. The emission intensity depends on the degree of exposure of the two tryptophan side chains to polar solvent and also on its proximity to specific quenching groups, such as protonated carbonyl, protonated imidazole, deprotonated ε-amino groups, and tyrosinate anions. The quenching of emission intensity of albumin was observed in the presence of cluster because of possible changes in the protein’s secondary structure, leading to changes in the tryptophan environment of albumin. We have measured the Stern-Volmer quenching constants at various temperatures. These results show that the quenching constant does not increase with higher temperature, indicating the static quenching mechanism (Table 1). Further, the higher slope for cluster in the plot of F/F_0_ versus cluster concentration (Figure 5), reveals the stronger protein-binding ability of the cluster with enhanced hydrophobicity.

### 2.4. Antiviral Activity

**Figure 6:**
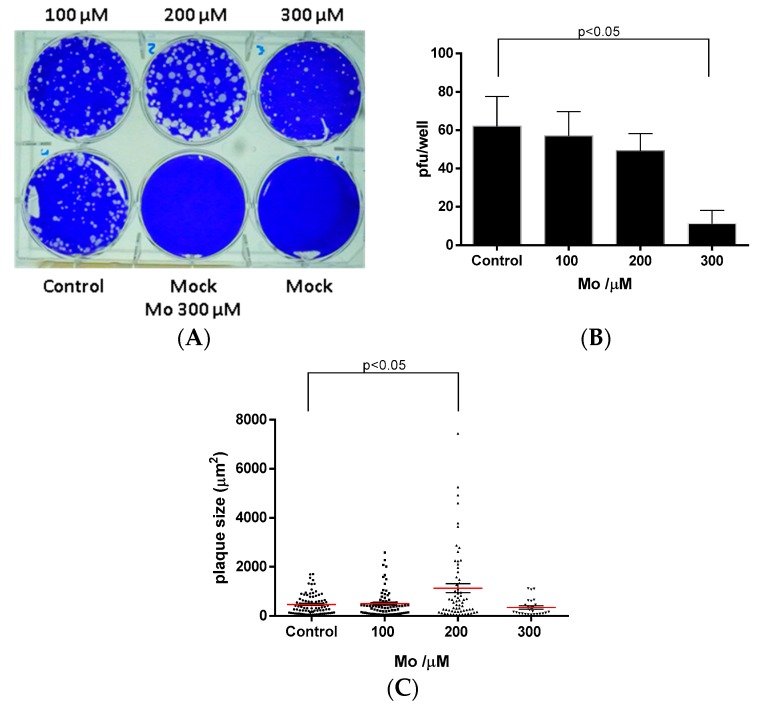
Effect of Mo cluster on plaque formation by rotavirus in MA104 cells. (**A**) MA104 cells were infected with rotavirus, treated with 100–300 µM of Mo cluster and incubated at 37 °C for 72 h; (**B**) Quantification of rotavirus plaque formation units (pfu). The figure shows the pfu/well corresponding to the mean (+/−SEM) of three independent experiments; (**C**) Quantification of rotavirus size plaque. The figure shows the pfu size corresponding to three independent experiments. Statistical analysis was performed by ANOVA test.

Results of MA-104 cells treatment with Mo-cluster showed that 300 μM could decrease the number of rotavirus SA11 plaque formation by 82 ± 6.8% when compared to the non-treated cells (control). This inhibitory effect was Mo-cluster dose-dependent (Figure 6A,B). Surprisingly, 200 µM of Mo-Cluster showed an effect in the foci-diameter (1133 ± 184 µM), while that 300 μM of Mo-cluster showed foci reduced slightly (349 ± 68 µM) compared with non-treated cells (470 ± 42 µM, see Figure 6C). These results showed that Mo-cluster has some specificity of action and supports the further investigation of this cluster for their potential as new antiviral compounds. Similar compounds have previously shown potential for antiviral [24], antibacterial [42], and antiparasitic [12] activity, increasing the potential application of molybdenum clusters in different areas of infectious disease research.

Biological properties of hexanuclear clusters have been previously described [11,15,17,43,44]. Among them, hexanuclear metal clusters have shown radiopacity under X-ray computed tomography [11], antitumoral properties [13], and cellular labelling [13,14,15,40] properties with low cytotoxicity. Hexanuclear clusters also have shown photosensitizer properties and are cytotoxic only under photoirradiation [29,40].

A final issue in biological safety is the potential of metal based drugs to induce a damaging immune response. Some metal based drugs have been reported as immunogenic in vitro, showing mainly innate immune response, evaluated on monocyte culture and proteomics analysis [45], and there have been few studies in vivo [46,47]. No reports on the immunogenicity associated to Molybdenum cluster exposition in humans have been published. In parallel, chronic exposition to molybdenum-containing food have shown toxic effects in ruminants [48] and humans [49], mainly associated to a decrease in copper absorption when molybdenum is present, but no immunological response in these studies has been reported. Possible mechanisms explaining a lack of immune response could be associated to a reduced aggregation of clusters in the presence of organic components [50,51]. In this study, potential immunogenicity induction of the cluster was not evaluated, but, considering the potential of metal based drugs to induce immune response, experiments evaluating immunological response in vitro are needed.

A biomarker is an indicator of a biological state of disease. It is characteristic of a specific state and, therefore, can be used as a marker for a target disease [52]. These biomarkers can be used to study cellular and subcellular processes [13] and monitor or recognize disruptions or alterations in the cellular processes of cancer cells. Biomarkers, specifically cancer biomarkers, are an indication of cancer and by detecting them, the existence of that specific cancer can be verified [53]. The potential biomedical application of luminescent clusters requires the evaluation of cellular uptake, subcellular localization, cytotoxicity, and biocompatibility in vitro [13,54]. This report describes the use of clusters as a specific biomarker agent against tumor cells. We also report the cellular uptake of the luminescent cluster and its subcellular distribution (Figure 1); based on its fluorescent properties, the cluster may be useful for cancer diagnostics, localization of tumors, and may enable the observation through fluorescence of tumor regression during treatment. Finally, we report the antiviral activity of the cluster, which is an interesting role to explore.

## 3. Materials and Methods

### 3.1. Preparation of [Mo_6_Cl_14_]^2−^ via Reduction of MoCl_5_ with Bismuth

Preparation of [Mo_6_Cl_14_]^2−^ was previously reported [55]. Briefly, an ampule with MoCl_5_ (5.0 g, 18.3 mmol) and Bi (3.825 g, 18.3 mmol) in the end reaction chamber was placed in the center of a horizontal tube furnace and the temperature slowly raised to 230 °C over 2 h and then to 350 °C over 2 h. The ampule was repositioned with part of the receiver chamber out of the furnace in order to remove BiCl_3_ by sublimation. Heating was continued at 350 °C for 24 h. After cooling, the ampule was opened in the glovebox. The crystalline, inhomogeneous nonvolatile was dark brown with areas of yellow and weighed 4.065 g. This weight was consistent with appreciable bismuth content since the theoretical yield of Mo_6_Cl_12_ was 3.053 g. A portion of the product (2.587 g) was treated with 25 mL of HCl 12 M with agitation, resulting in a slight exothermic reaction. The mixture was dissolved with heating and recrystallized as previously described, resulting in 1.025 g of orange-yellow needles of (H_3_O)_2_[Mo_6_(l_3_-Cl)_8_Cl_6_](OH_2_)_x_ (58% yield, for x = 6, based on MoCl_5_).

In a somewhat more labor-intensive procedure resulting in improved yield, MoCl_5_ (5.00 g, 18.3 mmol) and Bi (3.825 g, 18.3 mmol) were sealed in the receiver chamber of an ampule. The ampule was placed in the center of a horizontal tube furnace, the temperature slowly raised to 230 °C over 2 h and then to 350 °C over 2 h. The ampule was heated at 350 °C for 2.5 days. After cooling to 100 °C, the end of the receiver chamber was moved out of the furnace. The furnace was heated to 350 °C over 2 h and then at 350 °C for 12 h. The ampule was allowed to cool and then removed from the furnace. The yellow-brown nonvolatile material was homogenized by shaking, the ampule returned to the center of the furnace, and the ampule heated to 350 °C over 3 h.

The end of the receiver chamber was moved out of the furnace, the furnace reoriented to a slight angle (10–15°) from the horizontal, and the ampule heated at 350 °C for 24 h. The ampule was allowed to cool and was opened in the glovebox. The homogeneous, crystalline olive green/brown nonvolatile weighed 4.066 g (theory for Mo_6_Cl_12_, 3.053 g). A portion of this solid (2.000 g) was treated with HCl 12 M as previously described, and recrystallized to yield 1.456 g of orange-yellow chloromolybdic acid, (H_3_O)_2_[Mo_6_(l_3_-Cl)_8_Cl_6_](OH_2_)_x_ (80% yield, for x = 6, based on MoCl_5_) [55].

### 3.2. Cell Culture

The HepG2 hepatocellular carcinoma cell line (HB 8065; American Type Culture Collection), derived from a human hepatoblastoma (1) was maintained in Dulbecco’s modified Eagle’s medium (DMEM)-high glucose (GIBCO) with 10% heat-inactivated fetal bovine serum (FBS) (Gibco, Life Technologies, NY, USA) and antibiotic-antimycotic (Gibco, Life Technologies, NY, USA). Human umbilical vein endothelial cells (HUVEC)-derived endothelial cell line (EA.hy926) were kindly providing by C-J Edgell (2) and were grown in DMEM-low glucose (GIBCO) supplemented with 10% heat-inactivated FBS, antibiotic-antimycotic (Gibco, Life Technologies, NY, USA). All cell cultures were grown at 37 °C in a 5:95% CO_2_:air atmosphere. MA104 cells were maintained in DMEM (Gibco, Life Technologies, CA, USA) supplemented with 10% FBS (Gibco, Life Technologies, NY, USA) and antibiotic-antimycotic (Gibco, Life Technologies, NY, USA) at 37 °C and a 5% CO_2_ atmosphere.

### 3.3. Cell Viability Assay

Cell viability was evaluated using the 3-(4,5-dimethylthiazol-2-yl)-2,5-diphenyltetrazolium bromide (MTT) colorimetric assay (Invitrogen, Eugene, OR, USA). Cell viability was quantified by the amount of MTT reduction [56]. HepG2 and EA.hy926 cells were exposed to different concentrations of the cluster (0.1, 1, 10, 50, 100 and 250 µM) for 48 h. After treatment, cells were co-incubated with MTT (0.5 mg mL) for 4 h, and then solubilized with an acidified (HCl 0.04 N) isopropanol/dimethyl sulfoxide (DMSO) solution. Optical density was measured at 540 nm. All experiments were performed as triplicate. Data were expressed as percentage of survival cell, compared to the control.

### 3.4. Epifluorescence Microscope in Live Cells

HepG2 and EA.hy926 cells cultured in 6-well plates were exposed to different concentrations of the cluster (50–100 µM) during 24, 48 and 72 h. Then, cells were washed twice with phosphate buffered saline (PBS) and visualized using a microscopy EVOS^®^ FLoid^®^ cell (Life Technologies, Carlsbad, CA, USA).

### 3.5. Human Blood Samples and Preparation of Red Blood Cells

Blood samples were obtained from human voluntary donors. Donors agreed to allow the use of their blood in a hemolysis study by signing an informed consent. The study was approved by the local bioethics committee. Blood samples were obtained and prepared according to a protocol described before [16]. Briefly, blood samples obtained through venipuncture were anticoagulated, red blood cells were isolated by centrifugation and washed three times with phosphate-buffered saline (PBS; NaCl 150 mM, NaH_2_PO_4_ 1.9 mM, Na_2_HPO_4_ 8.1 mM, pH 7.4). Red blood cells were used immediately after isolation.

### 3.6. Analysis of Red Blood Cells Morphology

A sample of red blood cells was obtained from the incubated tubes, dropped in a glass, and stored in a humid chamber until observation under a microscope. Cells and red blood cells were visualized under a Motic AE-31 microscope station and photographed. Size of red blood cells was measured using ImageJ (Bethesda, MD, USA); morphological changes were evaluated by an expert in red blood cells morphology.

### 3.7. Test of Hemolysis

Red blood cells (hematocrit, 1%) were exposed to increasing concentrations of the cluster (10, 25, 50, 100 and 200 µM) in PBS. Red blood cells (hematocrit, 1%) suspended in distilled water or PBS were considered as controls for 100% or 0% of hemolysis, respectively. Hemolysis protocol was performed as previously reported [16]. Hemolysis was determined measuring the absorbance of supernatants at 540 nm immediately or after 24 h of incubation. A sample of red blood cells from the experiments were observed under an inverted microscope.

### 3.8. Albumin Interaction Studies

Quenching of the tryptophan residues of albumin was performed using the cluster as quencher. To solutions of human albumin in phosphate buffer at pH 7.4, increasing amounts of quenchers were added, and the emission signals in the range of 300–400 nm (excitation wavelength at 282 nm) were recorded after each addition of the quencher.

### 3.9. Anti-Rotavirus Activity

Antiviral activity was tested using a plaque lysis assay. MA104 cells were seeded on 6-well plates with Dulbecco’s Modified Eagle Medium (DMEM) gibco^®^ for 24–48 h until reaching ~100% of confluence. Before infection, rotavirus SA11-4F was activated with 10 µM/mL trypsin for 30 min at 37 °C. Cells were washed twice with PBS and were infected with 6 × 10^4^ pfu/mL for 1 h at 37 °C. A solution of MEM 2× with 1.4% agarose and 2.5 µg/mL trypsin with different concentrations of Mo-cluster (100–300 µM) were added. After 72 h of incubation, cells were fixed with 3.7% formaldehyde for 1 h and stained with 20% crystal violet solution for 30 min. Plaque lysis formation and size were analyzed with ImageJ software.

### 3.10. Data Analysis

These data represent the average ± SEM of at least three independent experiments performed in triplicate. Student’s *t*-test (Mann-Whitney) and ANOVA were used and considered significant at *p* < 0.05.

## 4. Conclusions

We observed that human cells treated with the molybdenum cluster reduced cell viability only following 72 h of incubation and caused minimal hemolysis in vitro even at high doses. The cluster also interacts and forms complexes with albumin. Furthermore, it showed anti-rotavirus activity. Based on these results and its fluorescent properties, the cluster could be useful for cancer diagnostics, localization of tumors, and may enable the observation through fluorescence of tumor regression during treatment. Furthermore, the cluster could be a promising antiviral agent.

Thus, the cluster [Mo_6_Cl_14_]^2−^ is a promising fluorescent component and could be studied in animal models at therapeutic doses for potential use in tracking cancer cells in vivo and as an antiviral agent.

## Figures and Tables

**Table 1:** Stern-Volmer dynamic quenching constants for the molybdenum [Mo_6_Cl_14_]^2−^ clusters and albumin. R is the correlation coefficient.

T (°K)	Ksv (L mol^−1^)	R
**298**	0.12	0.89
**310**	0.14	0.93
